# Cerebral Sinus Thrombosis: A Fatal Neurological Complication of Ulcerative Colitis

**DOI:** 10.4061/2010/132754

**Published:** 2010-04-29

**Authors:** Rodolfo Jose Nudelman, Daniel Gustavo Rosen, Emilie Rouah, Gordana Verstovsek

**Affiliations:** ^1^Department of Pathology, Baylor College of Medicine, 1 Baylor Plaza, Houston, TX 77025, USA; ^2^Department of Pathology, Michael E. DeBakey VA Medical Center, 2002 Holcombe Blvd, Houston, TX 77030, USA

## Abstract

Cerebral sinus thrombosis has been reported as an uncommon complication of ulcerative colitis (UC), occurring in up to 7.5% of cases. It is suspected to be a consequence of genetic predisposition and the hypercoagulable state occurring during disease relapse. We report a case of a 23-year-old male patient with one-year history of UC. He presented to the Emergency Room with left-sided progressive hemiparesis, numbness, hemiparesthesia, and pain, which followed a recent exacerbation of UC. The patient died 3 days after admission and an autopsy revealed superior and inferior sagittal sinus and cortical vein thrombosis with associated cerebral edema, hemorrhagic infarction, and herniation. The gastrointestinal tract had continuous cobblestone appearance extending from rectum to cecum, with hemorrhage and ulceration, consistent with active UC. Awareness of this rare complication of UC can contribute to early recognition and attempts at treatment of this serious and often fatal condition.

## 1. Introduction

Inflammatory bowel disease (IBD) comprises two major entities: ulcerative colitis (UC) and Crohn disease (CD). Ulcerative colitis is an idiopathic chronic IBD that is a consequence of complex interaction of environmental factors and genetic susceptibility [1]. It often occurs in patients between the ages of 20 and 30 years, with a second peak between the ages of 70 and 80 years. UC can be regarded as a systemic disease, and extraintestinal complications have been described in the skin, joints, bones, lungs, blood, eyes, kidneys, liver, and peripheral and central nervous system. CNS manifestations are particularly severe: they include thrombotic and cerebrovascular disease, myelopathy, cerebral vasculitis, multiple sclerosis, and acute disseminated encephalomyelitis [2]. Patients with IBD can also develop a hypercoagulable state and have been noticed to have an increased risk of various thrombotic events. These patients tend to suffer thrombosis earlier in life than patients without IBD [3]. Deep venous thrombosis and pulmonary thromboembolism are the two most common thrombotic complications of UC [4]. Cerebral vein and sinus thrombosis (CVST) has been reported as an uncommon but severe complication of UC and CD, ranging in frequency from 1.3% up to 7.5% of cases yearly depending on the clinical study [5]. The exact pathogenesis of this complication remains unknown and has been largely attributed to a hypercoagulable state that can occur in these patients. The thrombosis occurs more frequently during disease relapse but has also been reported 10 years after colectomy for UC [6]. The outcome is variable and may include either significant neurological deficit or death when not appropriately treated. Due to its nonspecific clinical presentation and low incidence, CVST is not usually readily recognized and treatment may be delayed, impacting on the prognosis. Neurology experts recommend prompt systemic anticoagulation, preferably with low-molecular weight heparin, and management of the acute complications of intracranial hypertension. Thrombolytics may be considered for those patients with rapid neurologic deterioration [7–19].

While most published cases associating CVST, UC, and CD [3, 4, 6, 16, 18, 20–28] (Table 1) report good clinical outcomes, that is not always the case. We report the case of a 23-year-old Caucasian male with UC who succumbed to a massive superior and inferior sagittal sinus and cerebral cortical vein thrombosis followed by a large and fatal cerebral hemorrhagic infarct, which occurred in the setting of UC exacerbation.

## 2. Case History

The patient was a 23-year-old Caucasian male with one-year history of UC. His disease was controlled until two weeks prior to hospital admission, when he presented with bloody diarrhea and abdominal pain. At that time he was placed on prednisone 20 mg, mesalamine 400 mg TID, promethazine 12.5 mg prn, metronidazol 500 mg TID, and omeprazole 20 mg for the UC relapse. He developed a worsening “band like” dull headaches and neck pain but did not seek medical attention until a week later, when he came to the Emergency Room. At the time of presentation, he showed progressive left sided hemiparesis accompanied by numbness, paresthesis, and severe pain; he was admitted for further evaluation. He denied fevers, photophobia, visual changes, dysarthria, or dysphagia. Neurological examination revealed left-sided hyperesthesia, hemiplegia, clonus, and a positive Babinski sign. Pertinent laboratory findings included prolonged prothrombine time (PT) of 17.8 seconds (reference range: 12.0–14.7 seconds), with an INR at 1.5, and a normal PTT at 29.0 seconds (reference range: 22.8–33.5 seconds). White blood cell count was slightly elevated at 10.3 K/cmm (normal 3.5–10) with 80% neutrophils. Mild microcytic anemia was present. A spinal tap was performed and the cerebrospinal fluid showed red blood cells, high glucose of 113 mg/dl (normal: 40–70), and high protein of 259 mg/dl (normal 15–45). Liver function tests were within normal limits. Urine drug screen was negative for benzodiazepines, marihuana, cocaine, opiates, amphetamines, and barbiturates. Serum Toxoplasma titers were negative. Abnormal electrolytes included chloride 99 mmol/L (normal 100–111), potassium 2.8 mmol/L (normal 3.5–5), and calcium 8.1 mg/dl (normal 8.9–10.3). Albumin was low at 2.4 g/dl (normal 3.5–5). D-dimer and other laboratory markers of fibrinolysis were not ordered at the time. Since they would be positive in several conditions such as hemorrhage, thrombosis, disseminated intravascular coagulation, malignancy, liver failure, and tissue trauma, these tests would not help in differentiating between these possibilities.

**Figure 1:**
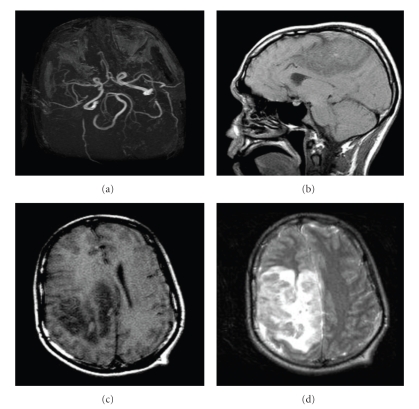
(a) MRI angiogram showing patent cerebral arteries. ((b), (c), (d)) Sagittal and transverse MRI views, T1 and T2 weighed, depict a large right hemorrhagic lesion with prominent edema and midline shift.

At the time of admission, CT and MRI images of the head were obtained (Figure 1). They showed rapidly progressive cortical and subcortical hemorrhages in the right frontal and parietal lobes with surrounding edema measuring up to 15 cm and midline shift, as well as unilateral enlargement of the left temporal region indicating impending uncal herniation. An MRI angiography of the head showed normal cerebral arteries with mass effect upon the right cerebral artery. A dedicated MRI venography was not performed. The differential diagnosis based on clinical and radiological findings included necrotizing vasculitis, an infectious process with secondary hemorrhage and thrombosis (such as toxoplasmosis, neurocysticercosis, and herpes encephalitis), cerebritis, primary and metastatic neoplastic processes, and cerebral venous thrombosis. The possibility of venous infarction or superior sagittal sinus thrombosis was considered, but the presentation was thought to be atypical given its unilaterality and the rapid progression of the lesion on imaging. Thrombosis was more strongly felt to be secondary to a primary infectious or vasculitic process. Treatment with steroids was instituted to decrease the mass effect and treat the most likely diagnosis, which was CNS vasculitis. Vancomicin, ceftriaxone, gancyclovir, acyclovir, and pyrimethamine were also started given the possibility of a bacterial, viral, or parasitic infectious etiology. No anticoagulation was administered due to the hemorrhagic nature of the lesion on imaging studies.

His clinical condition continued to worsen and mental status further deteriorated with development of additional neurological symptoms including hemianesthesia, slurred speech, anisocoria, and lethargy. The patient was transferred to the intensive care unit and was treated for increased intracranial pressure; therapeutic measures included induced hypocapnia, forced diuresis with IV-manitol, and placement of a ventriculostomy catheter. However, despite aggressive therapeutic measures the patient died within two days following admission. A complete autopsy was performed.

**Figure 2:**
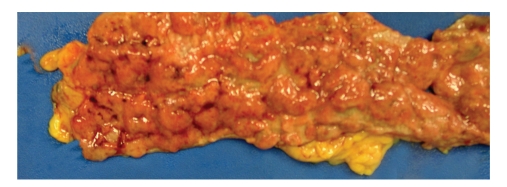
Gross photograph of the large bowel segment examined at autopsy. There is continuous cobblestone appearance of the mucosa, due to presence of multiple ulcerations and inflammatory pseudopolyps, a consequence of active ulcerative colitis.

**Figure 3:**
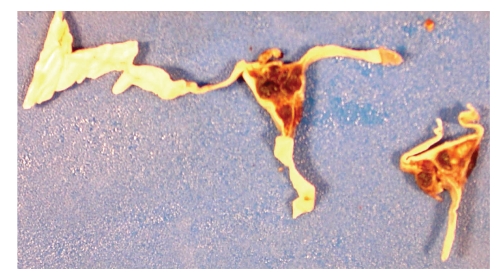
Gross photograph of dura and thrombosed superior sagittal sinus.

**Figure 4:**
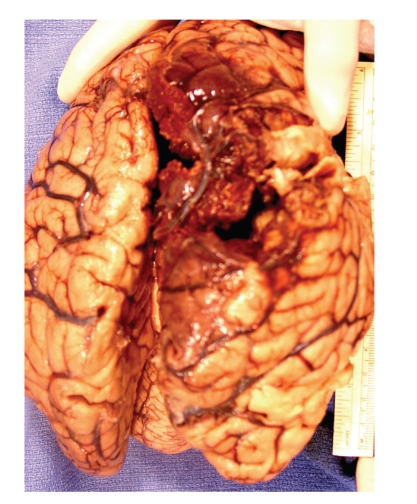
Gross photograph of brain showing right medial fronto-parietal hemorrhage and edema.

At autopsy, the gross examination of the gastrointestinal tract revealed continuous cobblestone appearance of the mucosa extending from the rectum up to the cecum, with areas of hemorrhage and ulceration (Figure 2). Microscopic examination revealed characteristic findings of UC, including chronic active colitis with crypt abscesses and inflammatory pseudopolyps. There were in addition multiple thrombi in the stomach, colon, liver, spleen, testis, and lung. CNS examination revealed superior and inferior sagittal sinus and cortical vein thrombosis (Figure 3), a right medial frontoparietal hemorrhagic infarct with cerebral edema (Figure 4), right cingulate, bilateral uncal and right cerebellar tonsillar herniation and Duret hemorrhages. Histological examination of the sections did not reveal any old infarcts or previous CNS pathology. Brain tissue was submitted for routine, acid fast, fungal, and viral cultures; all cultures were negative.

## 3. Discussion

We report a case of a 23-year-old male with one-year history of UC that developed superior and inferior cerebral sinus thrombosis following disease relapse. Despite aggressive therapy, the patient died two days following admission, an unusually rapid fatal outcome when compared to the other cases reported in the literature.

Thrombosis of the cerebral veins and sinuses usually affects young adults in their third decade of life with an annual incidence of 3 cases per 1 million population, although this varies widely depending on the study [2, 18, 29]. It also affects children, especially neonates with an incidence of 0.67 per 100,000 cases per year [30]. It accounts for less than 1% of all strokes [9]. Eighty-five percent of the patients show either an acquired or inherited prothrombotic risk factors at the time of presentation. A precipitating factor, such as reactivation of a chronic disease, can often be identified. The usual culprits include cancer, head trauma, drugs (especially oral contraceptives), hematologic diseases that result in hyperviscosity, systemic inflammatory diseases, systemic infections (especially sepsis) or infection involving the central nervous system, sinuses or ear, genetic susceptibility such as protein C and S deficiency and antithrombin deficiency, and other prothrombotic states such as pregnancy [1]. Our patient developed thrombosis of the cerebral veins in association with relapse of UC. A thorough personal and family medical history did not reveal any other prothrombotic risk factors, or a bleeding diathesis.

The etiology of hypercoagulation and thromboembolism in IBD remains poorly understood. Coagulation factor abnormalities such as elevated fibrinogen level, factor V, factor VIII, increase in circulating thrombin-antithrombin complexes, and decreased antithrombin III have been described; thrombocytosis and increased platelet aggregation have also been documented [3, 31, 32]. However, there is no substantial evidence to correlate hematological and coagulation abnormalities with cerebral sinus thrombosis. In our case, the only coagulation abnormality detected included prolonged PT, while aPTT, INR, and platelet count were normal. Whether other coagulation factors were abnormal and played a role in pathogenesis remains unknown.

A high level of suspicion of CVST is necessary in any patient with recent unusual headache, stroke like symptoms, seizures, or any other brain syndrome, due to its highly variable presentation. That suspicion should be even stronger in a young adult with neurological signs in the absence of the usual cerebrovascular risk factors, such as hypertension, diabetes, hyperlipidemia, smoking history, and prior history of cardio or cerebrovascular events. The most frequent sign at presentation is headache, which gradually increases over a period of days, although it can also present as an abrupt severe headache which can mimic subarachnoid hemorrhage [1]. Our patient did present with what he described as a band-like headache for a week before seeking medical attention. Seizures are very common, occurring in 40 percent of the cases; however they were not present in this case. Bilateral neurologic signs are the most common presentation, and they usually follow a sudden onset. While focal unilateral neurological symptoms such as aphasia or hemiparesis are less common, they were among early symptoms in our case. Symptoms from the other hemisphere usually follow after a few days. Thrombosis of the cerebral deep venous systems may present with other nonspecific signs, such as delirium, mutism, behavioral problems, or amnesia. When large lesions go unnoticed and therefore treatment is not instituted, cerebral herniation and death can occur. The delay from the onset of symptoms to diagnosis averages 7 days [33]. The most frequently involved vessels are transverse sinus (86%), superior sagittal sinus (62%), straight sinus (18%), cortical veins (17%), vein of Galen, and internal cerebral veins (11%) [1]. It is important to note that this particular patient had symptoms for at least a week before consulting a physician, which might have added to the unusual presentation of unilateral symptoms, leading to delay in diagnosis. Once CT and MRI images of the CNS were obtained, the hemorrhagic lesion was already too large, which led to a long list of differential diagnoses.

Once cerebral vein thrombosis is clinically suspected, an MRI and a magnetic resonance angiography (MRA) should be ordered, which will show an abnormal signal both in T1- and in T2-weighted images in a sinus or vein and absence of flow, respectively. CT is also helpful in ruling out other acute cerebral disorders and should be the first study ordered in an emergency setting. MRI and MRA are considered the best tools for diagnosis and follow-up [9].

Treatment of this disorder includes appropriate measures to prevent or reverse cerebral herniation and stabilize the patient. So far, acute management relies on treatment of acute intracranial hypertension. Corticosteroids are usually considered as a supplement to acetazolamide in patients who present with severe papilledema and are administered with the goal of reducing the systemic inflammatory state associated with UC relapse. Placement of a cerebrospinal fluid draining catheter may be necessary if medical management of intracranial hypertension is not helpful. All of these measures were taken in this case, but regrettably the rapid progression of the edema and herniation led to the patient's demise.

Several treatment modalities using different anticoagulation approaches have been evaluated, and studies and single-case reports on this subject are available for review (Table 1). Anticoagulant therapy is recommended to promote spontaneous thrombus resolution, circumvent thrombus extension, and prevent thromboembolism [9]. Most specialists now agree to institute treatment with heparins as soon as the diagnosis is confirmed by MRI or other imaging techniques, even in the presence of a hemorrhagic infarct [7, 9, 12, 17, 30]. One major concern with heparin treatment is the risk of hemorrhage in patients with ischemic infarcts. In the case we present, the extent of the hemorrhage was of such magnitude that neurologists and neurosurgeons decided to withhold the anticoagulant treatment since the risks of further bleeding into the CNS outweighed the possible benefits. The treatment was therefore based on measures to reduce the intracranial pressure.

For patients with ischemic infarcts due to cerebral vein thrombosis, a study comparing low-molecular-weight heparin (LMWH) and placebo for 3 weeks concluded that there was no significant increased risk of hemorrhage with heparin [8]. In addition, treatment with subcutaneous LMWH caused less major bleeding than intravenous heparin and decreased the risk of pulmonary emboli [34]. Oral anticoagulation should follow heparin treatment for at least 6 months, with a target INR of 2.5. Additional studies are needed to define the most effective treatment for this condition.

In summary, our patient presented with symptoms a week before seeking medical attention which led to a marked delay in his diagnosis and hence treatment. The unilateral presentation made the accurate suspicion for the diagnosis more difficult. The rapid progression of intracranial hemorrhage and hemorrhagic nature of the lesion precluded the use of anticoagulant therapy even though CVST was in the differential diagnosis and led to the final fatal outcome within two days of admission.

## 4. Conclusions

Cerebral sinus and vein thrombosis is a serious and often fatal complication of idiopathic inflammatory bowel disease if undiagnosed. It should be considered in any patient with little or no known vascular risk factors presenting with a severe headache and other focal or diffuse neurological signs. Appropriate clinical information, prompt neuroimaging, early diagnosis, and treatment are essential steps in avoiding potentially fatal outcome. It is our goal to raise the awareness and the index of suspicion among health professionals about this entity, as quicker diagnosis and prompt management of this complication are the only hopes to avoid fatal outcome.

## Figures and Tables

**Table 1:** Inflammatory bowel disease cases complicated by cerebral thrombosis: treatment and outcomes as reported in the reviewed literature.

Reference	Age/Sex	Inflammatory bowel disease	Onset of symptoms to diagnosis	Initial Treatment	Later Treatment	Outcome
Prasad et al. [16]	5/F	Crohn's	3 days	Venous sinus angioplasty and local tPA	Systemic anticoagulation	Complete recovery
Philips et al. [15]	35/F	UC	4 day	Local Urokinase and mechanical clot maceration	Systemic anticoagulation	Complete recovery
Philips et al. [15]	14/F	IBD	Unknown	Local Urokinase	Systemic anticoagulation	Complete recovery
Kao et al. [12]	14/F	UC	Unknown	Local urokinase	Systemic anticoagulation	Partial resolution of symptoms
Kao et al. [12]	7/F	UC	Unknown	LMWH	ASA	Partial resolution of symptoms
Kao et al. [12]	20/F	UC	Unknown	LMWH	Systemic anticoagulation	Complete recovery
Kao et al. [12]	13/F	UC	Unknown	LMWH	Systemic anticoagulation	Complete recovery
Wasay et al. [19]	23/F	UC	Unknown	Local Urokinase	Systemic anticoagulation	Partial resolution of symptoms
Wasay et al. [19]	34/F	UC	Unknown	Local Urokinase	Systemic anticoagulation	Complete recovery
Wasay et al. [19]	29/M	UC	Unknown	Local Urokinase	Systemic anticoagulation	Partial resolution of symptoms
Derdeyn and Powers [20]	26/F	UC	10	Warfarin	Systemic anticoagulation	Partial resolution of symptoms
Murata et al. [21]	19/M	UC	Unknown	Heparin	Unknown	Complete recovery
Samal et al. [22]	20/M	Crohn's	3	Oral anticoagulants	Unknown	Partial resolution of symptoms
Maag and Prayson [3]	30/M	Crohn's	1	Recanalization attempt	None	Death
Tsujikawa et al. [23]	27/M	UC	9	Heparin and Urokinase	Systemic anticoagulation	Complete recovery
Srivastava et al. [24]	29/M	UC	10	LMWH	Systemic anticoagulation	Complete recovery
Wartenberg and Palestrant [18]	43/F	UC	4	Local thrombolysis (rTPA)	Systemic anticoagulation	Partial resolution of symptoms
